# Clinical and self-perceived oral health assessment of elderly residents in urban, rural, and institutionalized communities

**DOI:** 10.6061/clinics/2019/e972

**Published:** 2019-08-13

**Authors:** Moan Jéfter Fernandes Costa, Caio Alano de Almeida Lins, Louise Passos Vigolvino de Macedo, Vanessa Patrícia Soares de Sousa, Jussaro Alves Duque, Marcelo Cardoso de Souza

**Affiliations:** I Programa de Pos Graduacao em Saude Coletiva, Faculdade de Ciencias da Saude do Trairi, Universidade Federal do Rio Grande do Norte, Santa Cruz, RN, BR; II Curso de Fisioterapia, Faculdade de Ciencias da Saude do Trairi, Universidade Federal do Rio Grande do Norte, Santa Cruz, RN, BR; III Faculdade de Odontologia, Universidade de Sao Paulo, Bauru, SP, BR

**Keywords:** Elderly Care, Quality of Life, Oral Health, Geriatric Dentistry, Public Health

## Abstract

**OBJECTIVES:**

This study aims to evaluate the self-perception of oral health according to the physical, psychosocial and pain/discomfort dimensions related to clinical conditions and orofacial pain of elderly people living in three different environments.

**METHODS:**

This was an observational, cross-sectional, quantitative study with a population-based approach and nonprobabilistic convenience sampling that included 81 elderly people: 27 resided in institutional homes for elderly individuals, 27 resided in an urban area and 27 resided in a rural area in the interior of Paraíba (PB) in northeastern Brazil.

**RESULTS:**

The Geriatric Oral Health Assessment Index (GOHAI) was used to assess self-perception of oral health, while the Questionnaire for Screening of Patients with Temporomandibular Disorders (QST/TMD) was used to assess the influences of orofacial pain and the biofilm indexes of teeth and prostheses. There was a statistically significant difference in the GOHAI scores among the places of residence, with the worst values associated with the rural area. According to the QST/TMD, the majority of individuals were affected by TMDs, with statistical differences for both sex and income.

**CONCLUSION:**

The biofilm analysis showed a higher incidence of clinical conditions in the rural population. The place of residence also influenced self-perception and the clinical oral health condition of elderly people; the rural population presented the worst results.

## INTRODUCTION

The health of elderly people is related to aspects inherent to general homeostasis and also to specific aspects such as problems related to the stomatognathic system. In this population, stomatognathic conditions are often associated with the period of time in which dental elements have been in the mouth, or conditions are due to the precariousness of the oral condition resulting from a heritage of curative and mutilating dentistry 1,2.

Understanding the housing conditions of the elderly population means analyzing the profile of the present social inequalities overall, as housing is related to quality of life and can influence the social level of communities 3.

The importance of evaluating the oral health component of general health is justified through indexes that evaluate how tooth loss, which is the main recurrent problem in this population, may be related to aspects of orofacial pain and healthy habits and lifestyles, as well as how it affects the quality of life 4-6.

Studies have shown that the oral health of institutionalized elderly people is often neglected due to a lack of preventive care or a lack of caregivers trained in this aspect. Additionally, the majority of elderly people, whether institutionalized or not, tend to dismiss oral health services because they believe that they no longer need/deserve this type of care, which aggravates their condition and their self-perception of health 4,5,7. This attitude may affect orofacial pain associated with lifestyle and habits and the quality of life 8,9.

Thus, the objective of this study was to evaluate the self-perception of oral health considering physical, psychosocial and pain/discomfort dimensions related to clinical conditions and orofacial pain in elderly people residing in three different environments.

## MATERIAL AND METHODS

This was an observational cross-sectional study with a quantitative population-based approach and nonprobabilistic convenience sampling among all the institutionalized elderly (n=27) people in a long-term institution for elderly individuals located in the city of Cuité, PB, in northeast Brazil; this is the only institution of this kind in the municipality. To ensure an comparable sample, the sample population consisted of all those institutionalized during the period of data collection, with the comparative sample consisting of residents of the community. The institutionalized sample population was matched by sex and age with elderly people residing in the urban (n=27) and rural areas (n=27) of the same municipality.

Elderly people who were able to understand and respond to the questionnaires and who were within the sex/age match parameters were included in the study. No participants from the institution were excluded from the sample; however, elderly people living in rural and urban communities who refused to sign the informed consent form were excluded and replaced so that the final samples from the three sites coincided, reducing the chance of selection bias.

The evaluations were carried out during a single individual interview with and intraoral dental examination of the elderly participants between July and August 2017 at a private location to avoid embarrassment and/or similarities between answers, which could represent a confounding factor and lead to some sort of memory bias.

First, data were collected from all the elderly people living in the institution. Data from the other sample populations were collected in the basic health units of several regions of the urban and rural zones of the same city.

The evaluation instruments used were as follows.

- Data regarding self-perception of oral health were collected with the Geriatric Oral Health Assessment Index (GOHAI) 10. The GOHAI enables a self-evaluation of oral health through 12 questions divided into the following dimensions: physical (chewing pattern), psychosocial (concern for oral health, satisfaction, dissatisfaction, appearance, self-awareness about oral health and social contact) and pain/discomfort (use of medication), with answers scored as 1, 2 and 3 (always, sometimes and never, respectively). Next, the 12 responses are added up to obtain the final score; relatively high scores (answers in the category “always” or number 1) indicate increased self-perception and good oral health, whereas relatively low scores indicate decreased self-perception and poor oral health conditions.
- Data related to orofacial pain were collected with the Questionnaire For Screening of Patients With Temporomandibular Disorders QST/TMD) 11. The questionnaire was translated, validated and summarized in Portuguese for the diagnosis and follow-up of TMDs; the questionnaire consisted of 5 questions with three answers for “always”, “sometimes” and “never”, for which the values 3, 2 and 1 were assigned, respectively. The questions were related to situations attributed to pain, discomfort or changes in the temporomandibular joint and face. The patient was classified as having temporomandibular disorder (TMD) when the sum was between 7 and 15, and the patient was classified as not affected by TMD when the sum was between 5 and 6.
- Data regarding the clinical condition of oral health were collected during an intraoral dental examination in which the teeth and prostheses were stained with basic fuchsin. For the dental elements, the index proposed by Silness and Loe 12 was implemented, which classifies the teeth according to a score (0 - without biofilm, 1 - a thin layer of biofilm, 2 - biofilm from the gingival region to the middle-third of the tooth, and 3 – biofilm abundant after examination of the four regions (vestibular, distal, mesial and lingual) of the teeth under study (16, 12, 34, 36, 32 and 44). The teeth that were missing were not replaced. The scores were summed and divided by the number of examined teeth by assigning final values of 1 when the mean of the values was between 0 and 0.5 (biofilm absent), and 2 when the mean was between 0.6 and 3 (biofilm present). The biofilm index for dentures, as proposed by Ambjornsen et al. 13, produces scores (0 - no biofilm, 1 – 0% to 25% of the regions covered in biofilm, 2% - 25 to 50% covered in biofilm, 3 – 50% to 75% covered in biofilm, and 4 – 75% to 100% covered in biofilm) for 5 delimited regions (incisive papilla and two maxillary tuber and two lateral regions). For the purposes of analysis, the value 1 was assigned when the sum was between 0 and 2 (biofilm absent), and 2 was assigned when the sum was between 3 and 20 (biofilm present).

The collected data were analyzed by descriptive and inferential analyses using the Statistical Package for Social Sciences (SPSS) software version 20. Normality tests were performed using the Kolmogorov-Smirnov test, which found that all variables had a nonnormal distribution. The descriptive analysis of the categorical variables was performed with frequencies.

The chi-square test was used to assess self-perceived oral health, the clinical examination findings and TMDs in relation to the places of residence. Fisher's exact tests were applied for the association analyses of these factors with the sociodemographic data due to a reduced sample size in some cases. A significance level of 5% (α<0.05) was established.

### Ethics

This study adopted Resolution 466/12 of the National Health Council (CNS) 14, which regulates research in humans, because it involved humans. This study followed the precepts of bioethics; it is registered in the National System of Ethics in Research and was submitted to and approved by the Research Ethics Committee (CEP FACISA/UFRN) (Order Number 2.116.337). All participants read and signed the informed consent form.

## RESULTS

The analysis of the results included the complete sample (n=81). Of the 81 participants, 63 (77.8%) were from the city of Cuité, PB; there was a predominance of females (n=60, 74.1%) and a higher prevalence of elderly people aged 60-65 years (32.1%). Of the total sample, 51.9% had an income of two to five minimum wages, and 54.3% lived with their partners. Detailed descriptions are presented in Table 1.

Table 2 presents the data regarding the association between the place of residence and the independent variables of the study. An association between place of residence and self-perception of health according to the GOHAI was found (*p*=0.004), with increased frequencies of good perception in the urban area and poor perception in the rural area (Table 2).

Table 3 shows the association between the QST/TMD score, GOHAI score, clinical conditions represented by biofilm on the teeth and prostheses and sociodemographic variables; there was a relatively greater difference among males (*p*=0.007) and among those with an income higher than 2 minimum wages (*p*=0.002).

It is interesting to point out that in Tables 2 and 3, regarding the variable biofilm, whether on the teeth or on the prostheses, the sample did not add up to n=27 in each group. Some participants had healthy teeth or prostheses and therefore were not clinically evaluated for this variable. Thus, only those participants who presented these conditions were included in this analysis, resulting in a reduced “n” in some cases.

## DISCUSSION

This cross-sectional study analyzed the perception of oral health, the clinical condition and orofacial pain associated with the sociodemographic factors and housing conditions of elderly people in three different environments: individuals living in institutions for elderly people and those living in their own homes in urban and rural areas.

According to the International Dental Federation 15, it is estimated that at least 50% of elderly people between the ages of 65 and 79 (target demographic) have at least 20 functional teeth in their mouth; this result was not observed in the present study. According to the biofilm index, which considered 6 index teeth, only 1/3 of the final sample (27 participants - visible in the dental biofilm index) had all of the evaluated teeth, reflecting the limited access to dental services and the curative and mutilating dentistry practices to which the great majority of this population has been subjected to throughout the course of their lives.

The self-perception of health can guide us and predict the need for care. Thus, it is believed that self-perception and clinical conditions of oral health are directly proportional. However, this phenomenon was not observed, as the values determined by the GOHAI suggested good self-perceived quality of oral health, although this was mainly found in the urban area (77.8%), which was considered a positive contributor. Additionally, we found that 75% (n=81) of elderly participants had positive biofilm evaluation indexes; 77.8% of rural residents and 84.6% of urban residents had biofilm present. This result was similar to many other studies that showed positive self-perception indexes but very poor conditions of clinical oral health. This can be observed in the SBBrasil 2010 project 16 and by Lima et al. 17, who found positive self-perception values associated with high edentulous indexes. In addition, another study 18 found that 75% of elderly people considered their oral health as excellent or good, but 95.7% of these people had only one or no teeth.

Studies have indicated that self-perception may contribute to the direction and planning of dental services 19,20, contrary to the results presented herein since self-perception was positive even in the presence of precarious clinical conditions. This may be because most elderly people (and also their caregivers and family members) tend to consider oral issues secondary to other systemic problems 9,21.

Emotional aspects related to housing and dignity are strongly associated with high satisfaction in the quality of life of elderly people 22. Thus, in observing the values from the GOHAI from residents in the institution, it was noted that the frequency (51.9%) of good self-perception of oral health quality exactly was directly associated with the care and comfort that the residents of this facility were receiving.

The GOHAI scores for self-perception of oral health were significantly different between three places of residence (*p*=0.004), with an increased frequency of “good perception” attributed to the urban zone (77.8%) and a increased frequency of “poor perception” attributed to the rural area (66.7%). A study 23 that compared rural and urban communities concluded that rural residents had poor rates of oral health due to lack of basic services and treatments regarding dental care.

Considering the precarious, curative and mutilating dentistry that elderly people have often been submitted to, most dental signs and symptoms may be overlooked, with pain and orofacial functionality also being disregarded. This was observed with the QST/TMD scores, in which the highest frequencies for all places of residence were attributed to individuals who were not affected to some degree by TMD, and there was significant difference in this regard. However, a significant difference was observed when sex and income were compared, with men reporting more conditions than women (*p*-value 0.007). On the other hand, people with a relatively higher income (from 2 to 5 minimum wages) were also more prone to be affected by TMD (*p*-value 0.002).

In relation to sex, it is clear that men were significantly more likely to be affected by TMD than women since men tend to seek out health services less than women, and when men do seek services, they are in a worse health state than women 24. Regarding income, it was observed 25,26 that income was a contributing factor to people seeking health services relatively more frequently; thus, diagnoses in these types of patients is easier because they access services more often than low-income patients.

One of the main limitations of this study was that it did not present cause-and-effect relationships between the conditions found. Due to the small sample size, inferences for larger populations cannot be made; however, the study was capable of clearly producing the proposed objectives.

Elderly residents of rural areas presented worse self-perceived and clinical oral health conditions than those living in urban areas and in a long-term institution.

Regarding orofacial pain, the place of residence did not influence the results; however, men and income between 2 and 5 minimum wages were associated with a higher prevalence of TMD.

## CONCLUSION

There was a significant difference between self-perception and the place of residence, with worse indexes associated with specific clinical components. Orofacial pain was not related to the place of residence; only income and sex were significantly associated with place of residence.

A cause-and-effect study is necessary to elucidate the conditions associated with vulnerability, especially in relation to the residents of the rural areas, where the worst indexes were found, with regard to both self-perceived oral health and clinical indicators.

## AUTHOR CONTRIBUTIONS

Costa MJF and Macedo LPV were responsible for data collection and manuscript writing. Lins CAA and Sousa VPS were responsible for statistical analyses. Duque JA was responsible for manuscript editing and review. Souza MC was responsible for guidance and manuscript final review.

## Figures and Tables

**Table 1:** Sociodemographic characteristics of the sample (n=81).

Variables	(%)
Age	
60-65	32.1
66-70	17.3
71-75	17.3
76-80	13.5
>80	19.8
Origin	
Cuité (PB)	77.8
Other locations	22.2
Sex	
Women	74.1
Men	25.9
Family income	
Up to 1 minimum wage	48.1
2 to 5 minimum wages	51.9
Marital status	
Without a partner	45.7
With a partner	54.3

PB - Paraíba

**Table 2:** Association of self-perceived oral health status, clinical condition and TMD with the place of residence.

Variables	Place of Residence			X^2^ value	*p*-value
	Urban Area n(%)	Institutional Homes n(%)	Rural Area n(%)		
GOHAI				10.846	0.004*
High perception	21 (77.8)	14 (51.9)	9 (33.3)		
Low perception	6 (22.2)	13 (48.1)	18 (66.7)		
QST/TMD				0.113	0.945
Affected	9 (33.3)	8 (29.6)	9 (33.3)		
Not affected	18 (66.7)	19 (70.4)	18 (66.7)		
OVERALL BIOFILM				4.160	0.125
No plaque	6 (22.2)	4 (50.0)	4 (15.4)		
Plaque present	21 (77.8)	4 (50.0)	22 (84.6)		
DENTAL BIOFILM				3.431	0.180
No plaque	1 (20.0)	4 (57.1)	3 (20.0)		
Plaque present	4 (80.0)	3 (42.9)	12 (80.0)		
PROSTHESES BIOFILM				3.197	0.202
No plaque	5 (22.7)	0 (0.0)	0 (0.0)		
Plaque present	17 (77.3)	1 (100.0)	11 (100.0)		

GOHAI - Geriatric Oral Health Assessment Index; QST/TMD - Questionnaire For Screening Of Patients With Temporomandibular Disorders; BIOFILM - index that evaluates the amount of bacterial plaque adhered to the teeth and total prostheses (TP);

* Statistical difference found for the place of residence in relation to GOHAI.

**Table 3:** Association of sociodemographic characteristics with self-perceived clinical oral health and TMD variables.

	Gohai n(%)			Gst/dtm n(%)			Dental Biofilm n(%)			Denture Biofilm n(%)		
	Low	High	*p*-value	Affected	Not affected	*p*-value	Present	Absent	*p*-value	Present	Absent	*p*-value
**Sex**			0.30			0.007*			0.20			1.0
Man	12 (32.4)	9 (20.5)		12 (46.2)	9 (16.4)		5 (62.5)	6 (31.6)		1 (20.0)	6 (20.7)	
Woman	25 (67.6)	35 (79.5)		14 (53.8)	46 (83.6)		3 (37.5)	13 (68.4)		4 (80.0)	23 (73.3)	
**Age**			0.06			0.869			0.58			0.47
60-65	9 (24.3)	17 (38.1)		10 (38.5)	16 (29.1)		6 (75.0)	8 (42.1)		3 (60.0)	7 (24.1)	
66-70	5 (13.5)	9 (20.5)		3 (11.5)	11 (20.0)		0 (0.0)	4 (21.1)		0 (0.0)	5 (17.2)	
71-75	5 (13.5)	9 (20.5)		4 (15.4)	10 (18.2)		1 (12.5)	2 (10.5)		2 (40.0)	7 (24.1)	
76-80	9 (24.3)	2 (4.5)		4 (15.4)	7 (12.7)		1 (12.5)	3 (15.8)		0 (0.0)	5 (17.2)	
>80	9 (24.3)	7 (15.9)		5 (19.2)	11 (20.0)		0 (0.0)	2 (10.5)		0 (0.0)	5 (17.2)	
**Income**			0.82			0.002*			0.41			0.15
1 Minimum wage	17 (45.9)	22 (50.0)		6 (23.1)	33 (60.0)		4 (45.0)	6 (31.6)		0 (0.0)	11 (37.9)	
2-5 Minimum wages	20 (54.1)	22 (50.0)		20 (76.9)	22 (40.0)		4 (50.0)	13 (80.4)		5 (100)	18 (62.1)	
**Ethnicity**			0.33			0.143			1.00			0.28
White	30 (81.1)	30 (68.2)		23 (88.5)	37 (67.3)		7 (87.5)	16 (84.2)		3 (60.0)	21 (72.4)	
Black	2 (5.4)	7 (15.9)		1 (3.8)	8 (14.5)		0 (0.0)	2 (10.5)		0 (0.0)	5 (17.2)	
Latino	5 (13.5)	7 (15.9)		2 (7.7)	10 (18.2)		1 (12.5)	1 (5.3)		2 (40.0)	3 (10.3)	

GOHAI - Geriatric Oral Health Assessment Index; QST/TMD - Questionnaire For Screening Of Patients With Temporomandibular Disorders. BIOFILM - index that evaluates the amount of bacterial plaque adhered to the teeth and total prostheses (TP);

* Statistical difference found for gender and income based on the QST/TMD.
